# Platelet-rich fibrin and concentrated growth factor in the treatment of immediate implants in teeth with periapical lesions: A clinical trial with two-year follow-up

**DOI:** 10.34172/joddd.025.41670

**Published:** 2025-06-30

**Authors:** Maryam Zohary, Meysam Malekzadeh, Ashkan Salari, Amin Afzali, Dina Maleki

**Affiliations:** ^1^Department of Periodontics, School of Dentistry, Guilan University of Medical Sciences, Rasht, Iran; ^2^Dental Sciences Research Center, School of Dentistry, Guilan University of Medical Sciences, Rasht, Iran

**Keywords:** Blood platelets, Bone regeneration, Dental implant, Platelet-rich fibrin

## Abstract

**Background.:**

This study was designed to compare the effect of platelet-rich fibrin (PRF) and concentrated growth factor (CGF) on the immediate implants placed in previously infected sites.

**Methods.:**

A total of 210 patients were included in this randomized clinical trial. The remaining tooth was extracted, and the periapical lesion was removed. Then, PRF and CGF were placed in the socket in the PRF and CGF groups, respectively. No intervention was performed in the control group. The implant was inserted and sutured. Postoperative pain was measured using VAS. Plaque index (PI), bleeding on probing (BOP), buccal and proximal gingival esthetic index (GEI-B and GEI-P), crestal bone level, periapical lesion size, and Implant Health Scale (IHS) were evaluated. Data were analyzed with SPSS 18 using one-way ANOVA, Tukey, and repeated measures test at 0.05 significance level.

**Results.:**

The mean BOP and pain in each period were significantly higher in the control group than in the PRF and CGF groups and in the PRF group than in the CGF group (*P*=0.001 and *P*<0.001). In the PRF, CGF, and control groups, pain had a decreasing trend (*P*=0.010, *P*<0.001, and *P*=0.001); PI had a significant increasing trend (*P*=0.034, *P*<0.001, and *P*<0.001); crestal bone level had a significant increasing trend (*P*=0.023, *P*=0.033, and *P*<0.001); and the size of periapical lesion had a significant decreasing trend (*P*=0.004, *P*=0.002, and *P*=0.048). The IHS showed optimum health after two years in the PRF and CGF groups. Optimum health, satisfactory survival, and compromised survival were reported in the control group.

**Conclusion.:**

PRF and CGF guaranteed the successful osseointegration of immediate implants in previously infected sites. CGF and PRF positively affected the soft tissue and hard tissue around the immediate implants. CGF had more promising effects than PRF.

## Introduction

 Dental implants replace lost teeth. Immediate implant placement was first introduced by Schulte et al. in 1978.^1,2^ According to previous studies, immediate and delayed dental implant placement have similar survival and success rates in clinical studies and similar healing patterns in histological studies.^3-5^ Immediate insertion of dental implants in extracted tooth sockets provides favorable advantages, such as reducing the total treatment sessions and decreasing patient discomfort due to fewer surgeries.^6,7^ However, immediate implants in sites with periodontal or endodontic infection are challenging as the infection may interfere with and reduce the osseointegration.^8,9^

 To overcome this limitation of immediate implants after extracting teeth with periodontal or endodontic infection and to improve osseointegration, autologous blood derivatives containing inflammatory mediators and growth factors can be used to enhance tissue healing.^10-12^ Platelet-rich plasma (PRP) was the first generation of platelet concentrates containing a high concentration of platelets and fibrinogen.^12-14^ The fibrin scaffold formed by the PRP activates the platelets in the PRP, leading to the release of essential growth factors.^13-15^ Platelet-rich fibrin (PRF), the second generation of platelet concentrates, exhibits osteogenicity and PRP properties.^12,15^ Concentrated growth factors (CGFs) are generated through a controlled centrifugation procedure and exhibit a much larger and denser fibrin matrix containing higher concentrations of growth factors.^16,17^

 Therefore, this study was designed to compare the effect of platelet-rich fibrin (PRF) and CGF on postoperative pain, periodontal health and esthetic, the level of the crestal bone, the size of the lesion, and the implant survival/success of immediate implants placed in previously infected sites during a two-year follow-up.

## Methods

###  Study design

**Figure 1:**
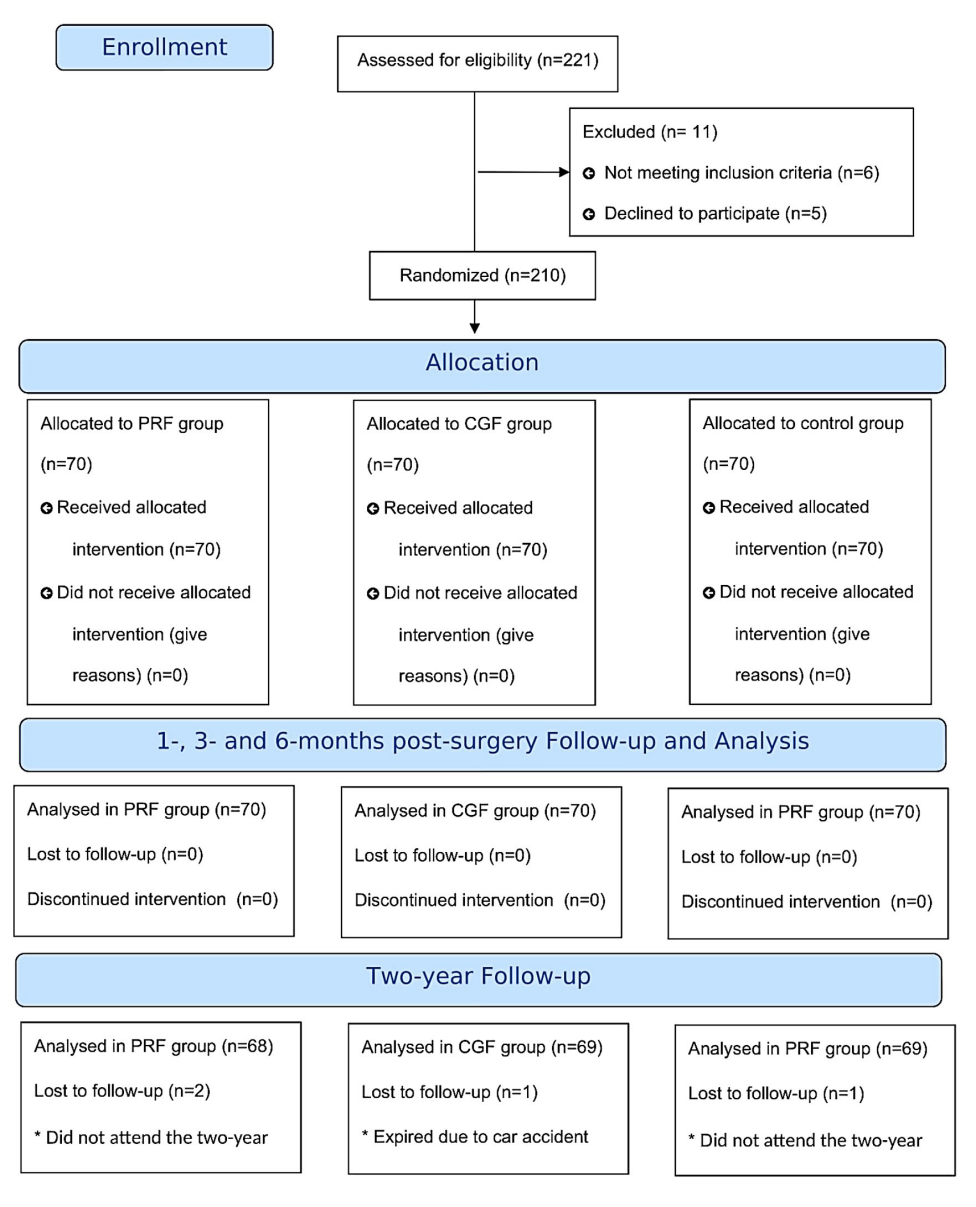
The flow diagram includes detailed information on the excluded participants

 A total of 210 patients requiring tooth extraction and immediate implant placement were included in this randomized clinical trial; 210 dental implants were assessed in this study. Ethical clearance was obtained from the Ethics Committee of Guilan University of Medical Sciences. All the participants agreed to participate by signing an informed consent. The eligibility criteria and trial method did not change during the trial. The study was carried out from January 2021 to January 2023. The methodology was designed according to the Consolidated Standards of Reporting Trials (CONSORT 2010) (Figure 1).

###  Eligibility criteria 

 Patients were included in the study if they were 20‒60 years old, had healthy systemic status, had a plaque index (PI) of < 25%, and had remaining unrestorable tooth or root with periapical lesion. Patients were excluded if they smoked, were pregnant or lactating, had a history of radiotherapy or chemotherapy, had bruxism, had pain, swelling, pus, abscesses, palatal plate perforation, type 2 or 3 sockets, advanced periodontitis, marginal bone loss, and had a periapical lesion > 7 mm.

###  Sample size calculation

 Considering three groups with means of 5.11 (PRF), 4.71 (CGF), and 2.91 (control), a statistical power of 0.84, a significance level of 0.05, and a standard deviation of 1.33 obtained from a previous study, the minimum sample size per group, accounting for a 10% dropout rate, was calculated at n = 70.^18^

###  Patient recruitment and group assignment

 The participants were selected from the Periodontics Department if they met the inclusion criteria and were randomly assigned to group A (PRF), group B (CGF), or group C (control) with an allocation ratio of 1:1:1 following the random permutation block method. Each patient was enrolled in only one study group, even if they had more than one tooth that met the inclusion criteria. Therefore, each dental implant represented one patient. A calibrated researcher, blinded to the allocation sequence, completed the baseline assessments.

###  Clinical examination

 Postoperative pain, periodontal health and esthetic, the level of the crestal bone, the size of the lesion, and the implant survival/success were reported in the current study. The pain was measured using a visual analog scale (VAS) 1, 6, 24, and 48 hours after surgery. Periodontal health was recorded using O’Leary PI and bleeding on probing (BOP) 1, 3, and 6 months after surgery. Periodontal esthetic was evaluated using gingival esthetic index-buccal (GEI-B) and gingival esthetic index-proximal at 1, 3, 6, 12, and 24 months after surgery. GEI-B measured the difference of the buccal gingival margin of the implant from the adjacent teeth and was reported as score 0 (no difference), score 1 ( < 1 mm), score 2 (1‒2 mm), score 3 (2‒3 mm) and score 4 ( ≥ 4 mm). GEI-P measured the condition of the papilla and was reported as score 0 (absence of papilla), score 1 (presence of papilla less than half of proximal embrasure), score 2 (presence of papilla more than half of proximal embrasure), score 3 (papilla filling the proximal embrasure) and score 4 (papilla growing over the proximal embrasure) 6, 12, and 24 months after surgery. The level of crestal bone to the implant platform and the dimension of the periapical lesion were measured using long-cone paralleling periapical radiography.

 The implant survival/success was assessed after 12 and 24 months using the Implant Health Scale (IHS) and reported as score 1 (optimum health; no pain and tenderness, no mobility, no exudate, and < 2 mm of bone loss), score 2 (satisfactory survival; no pain and tenderness, no mobility, no exudate, and 2‒4 mm of bone loss), score 3 (compromised survival; possible pain and tenderness, no mobility, a probing depth of ≥ 7 mm, possible exudate, and > 4 mm of bone loss), and score 4 (failure; the presence of pain and tenderness, mobility, probing depth of ≥ 7 mm, the presence of exudate, and bone loss more than the half of the implant length).

###  Intervention

 One hour before surgery, the patients received 1 g of amoxicillin and 325 mg of acetaminophen; 0.12% chlorhexidine and iodine were used before surgery. Local anesthesia (2% lidocaine HCl with 1:100 000 epinephrine) was injected, and the remaining tooth or root was extracted using the periotome curettes PT2 and PT3 (Hu-Friedy, Chicago, USA) and forceps with rotational motions. Using a Prichard periodontal surgical curette (Hu-Friedy, Chicago, USA), the remaining periapical tissue was removed, and the socket walls were checked to be intact. At this stage, no intervention was performed in the control group; CGF and PRF were placed into the fresh socket in the CGF and PRF groups, respectively. One surgeon performed all the surgeries.

**Figure 2:**
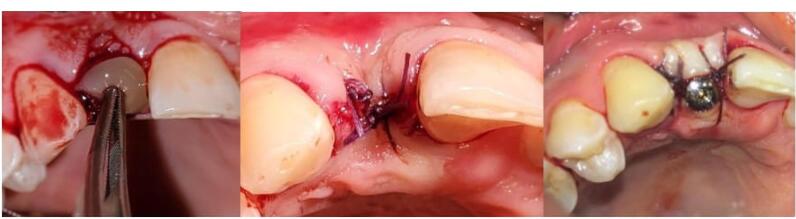
The placement of CGF in the socket; suturing of the surgical site; after placing the healing abutment

 Dental implants (Euroteknika, Sallanches, France) were placed 2‒3 mm apically than the gingival margin in all the patients. The minimum insertion torque for initial implant stability was 25‒30 Ncm. The transmucosal abutment was then inserted. In all the groups, the flap was partially displaced coronally, and a simple loop suture was performed using a 4-0 silk suture (Figure 2).

###  PRF preparation

 To prepare PRF, 20 mL of the patient’s blood was obtained. The blood-containing tubes were transferred to a centrifuge (Medifuge, Silfradent, Sofia, Italy) and centrifuged at room temperature at 2700 rpm for 2 minutes. PRF was removed from the other cells using sterile forceps and scissors and transferred to a sterile compress.

###  CGF preparation

 To prepare CGF, approximately 9 mL of the patient’s blood was collected in sterile tubes. The tubes were centrifuged (Medifuge, Silfradent, Sofia, Italy) for 2 minutes at 2700 rpm, 4 minutes at 2400 rpm, 4 minutes at 2700 rpm, and 3 minutes at 3000 rpm. CGF adhesive was taken from the tubes, and the two connected phases and the center and the bottom layers were separated with scissors. When the CGF adhesive was removed, some growth factor was at the interface between the CGF adhesive and erythrocyte layers. Therefore, a certain amount of red blood cells was retained during isolation to ensure the content of growth factors. CGF was compressed in molds to obtain a CGF membrane. CGF and CGF membranes were placed in sterile normal saline for use.

###  Statistical analysis 

 The data were analyzed using SPSS 18.0 (SPSS Inc., Chicago, IL, USA). ANOVA, post hoc Tukey tests, and repeated-measures ANOVA were applied. The significance level of 0.05 was set.

## Results

 A total of 210 patients participated in the current study. Data of all patients were analyzed for the first year of follow-up. However, the data of four patients were excluded for the two-year follow-up. Two patients from the CGF group and one from the control group did not attend the two-year follow-up. One patient from the PRF group had expired throughout the study. The mean age of patients was 48.28 ± 7.05; 47.6% (100) of participants were female, and 52.4% (110) were male.

Table 1 shows the mean pain of patients according to the study groups. Participants in the control group experienced significantly higher levels of pain than the PRF and CGF groups 1, 6, 24, and 48 hours after surgery. The patients in the PRF group experienced significantly higher levels of pain than the CGF group (*P* < 0.001, *P* < 0.001, *P* < 0.001, and *P* < 0.001, respectively). Pain significantly decreased during the first 48 hours after surgery in the PRF, CGF, and control groups (*P* = 0.01, *P* < 0.001, and *P* = 0.001, respectively).

**Table 1:** The mean of experienced pain 1, 6, 24, and 48 hours after surgery

**Pain**	**CGF**	**PRF**	**Control**
1 hour after surgery	4.42 ± 0.97	6.14 ± 0.69	8.28 ± 0.75
6 hours after surgery	3.57 ± 0.78	5 ± 0.57	7.57 ± 0.53
24 hours after surgery	3 ± 0.81	4 ± 0.81	4.85 ± 0.69
48 hours after surgery	2 ± 0.57	2.57 ± 0.53	3.42 ± 0.53

CGF: Concentrated growth factors PRF: platelet-rich fibrin

 The difference in PI between the control, PRF, and CGF groups was not significant at baseline and 1, 3, and 6 months after surgery (*P* = 0.745, *P* = 0.584, *P* = 0.592, and *P* = 0.685, respectively). PI significantly increased 6 months after surgery in the PRF, CGF, and control groups (*P* = 0.034, *P* < 0.001, and *P* < 0.001, respectively) (Table 2).

**Table 2:** The mean of PI at baseline and 1, 3, and 6 months after surgery

**PI**	**CGF**	**PRF**	**Control**
Baseline	16 ± 2.11	16.42 ± 2.14	16.9 ± 2.24
1 month after surgery	16.71 ± 2.28	16.85 ± 1.57	17 ± 1.43
3 months after surgery	18.71 ± 1.97	17.85 ± 2.26	18.85 ± 2.16
6 months after surgery	19.71 ± 1.25	19.42 ± 1.39	19.91 ± 1.29

PI: Plaque Index, CGF: Concentrated growth factors PRF: platelet-rich fibrin

 The mean of BOP was not significantly different between the three study groups at baseline and 1 and 3 months after surgery (*P* = 0.171, *P* = 0.125, and *P* = 0.169, respectively). However, 6 months after surgery, the mean of BOP was significantly higher in the control group than in the PRF and CGF groups and significantly higher in the PRF group than the CGF group (*P* = 0.001). Six months after surgery, BOP had a decreasing trend in the PRF and CGF groups (*P* = 0.003 and *P* = 0.043), with an increasing trend in the control group (*P* = 0.004) (Table 3).

**Table 3:** The mean of BOP at baseline and 1, 3, and 6 months after surgery

**BOP**	**CGF**	**PRF**	**Control**
Baseline	17.81 ± 2.54	17.85 ± 1.06	16.31 ± 1.11
1 month after surgery	16.42 ± 1.13	17.14 ± 0.69	15.07 ± 0.97
3 months after surgery	15 ± 0.81	15.71 ± 0.75	15.57 ± 0.53
6 months after surgery	14.42 ± 0.53	15.61 ± 2.11	19.57 ± 0.76

BOP: Bleeding on Probing, CGF: Concentrated growth factors PRF: platelet-rich fibrin

 GEI-B was not significantly different between the three study groups at baseline and 1, 3, 6, 12, and 24 months after surgery (*P* = 0.602, *P* = 0.267, *P* = 0.116, *P* = 0.106, *P* = 0.104, and *P* = 0.087, respectively). The differences in GEI-P between the control, CGF, and PRF groups were not significant at baseline and 1, 3, 6, 12, and 24 months after surgery (*P* = 0.487, *P* = 0.616, P = 0.824, *P* = 0.830, *P* = 0.961, and *P* = 0.966, respectively). Despite changes in GEI-B and GEI-P during the 24 months of the study, they were not significant in any of the study groups (Tables 4 and 5).

**Table 4:** The mean of GEI-B at baseline and 1, 3, 6, 12, and 24 months after surgery

**GEI-B**	**CGF**	**PRF**	**Control**	***P*** ** value**
Baseline	0.25 ± 0.48	0.28 ± 0.48	0.57 ± 0.78	0.602
1 month after surgery	0.11 ± 0.37	0.14 ± 0.37	0.57 ± 0.78	0.267
3 months after surgery	0	0.14 ± 0.37	0.57 ± 0.78	0.116
6 months after surgery	0	0.09 ± 0.37	0.57 ± 0.78	0.106
12 months after surgery	0	0.07 ± 0.23	0.52 ± 0.71	0.104
24 months after surgery	0	0.06 ± 0.5	0.47 ± 0.2	0.87
*P* value	0.304	0.061	0.138	-

GEI-B: buccal gingival esthetic indexCGF: Concentrated growth factors, PRF: platelet-rich fibrin.

**Table 5:** The mean of GEI-P at baseline and 1, 3, 6, 12, and 24 months after surgery

**GEI-P**	**CGF**	**PRF**	**Control**	***P*** ** value**
Baseline	1.85 ± 0.69	1.71 ± 0.75	1.42 ± 0.53	0.487
1 month after surgery	1.85 ± 0.69	1.71 ± 0.75	1.71 ± 0.75	0.616
3 months after surgery	2.71 ± 0.48	2.1 ± 0.37	1.28 ± 0.48	0.824
6 months after surgery	2.71 ± 0.48	2.42 ± 0.53	1.28 ± 0.48	0.830
12 months after surgery	2.8 ± 0.25	2.46 ± 0.12	1.22 ± 0.12	0.981
24 months after surgery	2.7 ± 0.21	2.44 ± 0.6	1.2 ± 0.13	0.966
*P* value	0.056	0.061	0.093	-

GEI-P: proximal gingival esthetic index, CGF: Concentrated growth factors, PRF: platelet-rich fibrin.

 The mean of crestal bone level to implant platform was not significantly different between the study groups at baseline and 6, 12, and 24 months after surgery (*P* = 1.000, *P* = 0.134, *P* = 0.337, and *P* = 0.467, respectively). During the 24 months of the study, the crestal bone levels increased significantly in the PRF, CGF, and control groups (*P* = 0.023, *P* = 0.033, and *P* < 0.001, respectively) (Table 6).

**Table 6:** The mean of crestal bone level at baseline, 6-, 12-, and 24- months post-surgery

**Crestal bone level**	**CGF**	**PRF**	**Control**	***P*** ** value**
Baseline	0	0	0	1.000
6 months after surgery	0.09 ± 0.5	0.05 ± 0.05	0.04 ± 0.05	0.134
12 months after surgery	0.14 ± 0.5	0.12 ± 0.1	0.09 ± 0.1	0.337
24 months after surgery	0.19 ± 0.5	0.16 ± 0.1	0.13 ± 0.1	0.476
*P* value	0.023	0.033	< 0.001	

CGF: Concentrated growth factors, PRF: platelet-rich fibrin.

 The mean size of the periapical lesion was not significantly different between the study groups at baseline and 6 and 12 months after surgery (*P* = 0.716, *P* = 0.276, and *P* = 0.117, respectively). However, 24 months after surgery, the periapical lesion was significantly smaller in the CGF group than the PRF and control groups and significantly smaller in the PRF group than the control group (*P* = 0.032). During the 24 months of the study, the mean size of the periapical lesion decreased significantly in the PRF, CGF, and control groups (*P* = 0.004, *P* = 0.002, and *P* = 0.048, respectively) (Table 7). Table 8 presents the results of IHS.

**Table 7:** The mean of periapical lesion size at baseline and 6, 12, and 24 months after surgery

**Peri-apical lesion size**	**CGF**	**PRF**	**Control**	***P*** ** value**
Baseline	2.75 ± 0.78	2.94 ± 0.37	2.85 ± 0.69	0.716
6- months post-surgery	2.71 ± 0.48	2.57 ± 0.53	2.63 ± 0.57	0.276
12- months post-surgery	1.28 ± 0.48	1.57 ± 0.53	2.2 ± 0.57	0.117
24- months post-surgery	0.3 ± 0.24	0.5 ± 0.37	1.9 ± 0.87	0.032
*P* value	0.002	0.004	0.048	

CGF: Concentrated growth factors, PRF: platelet-rich fibrin.

**Table 8:** IHS 12- and 24-month post-surgery

**IHS**		**CGF**	**PRF**	**Control**
12 months after surgery	Optimum health	100% (70)	100% (70)	84% (59)
	Satisfactory survival	0% (0)	0% (0)	16% (11)
	Compromised survival	0% (0)	0% (0)	0% (0)
	Failure	0% (0)	0% (0)	0% (0)
24 months after surgery	Optimum health	97% (68)*	98% (69)*	80% (56)*
	Satisfactory survival	0% (0)	0% (0)	11% (8)
	Compromised survival	0% (0)	0% (0)	8% (5)
	Failure	0% (0)	0% (0)	0% (0)

*Two patients from the CGF group and one from the control group did not attend the two-year follow-up. One patient from the PRF group had expired. HHS: Implant Health Scale, CGF: Concentrated growth factors, PRF: platelet-rich fibrin.

## Discussion

 One of the primary goals of implant treatment is to restore function and improve beauty.^1,3^ Several studies have examined the placement of immediate implants in the socket of a newly extracted infected tooth. However, few studies have evaluated the effect of PRF and CGF on the success of this treatment.^19-23^ Periapical infection is a potential risk factor for implant success because anaerobic pathogens in the area can infect the implant and lead to peri-implantitis.

 In the current study, PI significantly increased in all study groups; however, the mean of PI was not significantly different between them. Medikeri et al^19^ assessed the effect of PRF on the PI. Similar to the current study, PI increased by 3, 6, and 12 months after the implant placement for 34%, 39.16%, and 48.37%, respectively. Medikeri et al^19^ also reported no significant differences between the PRF and control groups regarding PI. Isler et al^20^ studied the changes in PI in the CGF and control groups. In agreement with the current study, Isler et al^20^ reported that PI increased from 0.49 to 0.67 in the CGF group 6 months after surgery. Isler et al^20^ also found no significant differences between the CGF and control groups. The findings of this study and the studies of Medikeri et al and Isler et al were consistent. PI is an index directly affected by patients’ plaque control. As time passes, patients may not follow the oral hygiene protocols properly; therefore, the index increased throughout the studies.

 In this study, BOP significantly decreased in the PRF and CGF groups, with a significant increase in the control group. Medikeri et al reported that BOP significantly decreased during the 12-month study period in the PRF group. Isler et al^20^ claimed that BOP and gingival index significantly decreased in the CGF group during the first 6 months after surgery (from 97.12 to 20.19 and 1.12 to 0.36, respectively). Consistent with the current study, Medikeri et al^19^ and Isler et al^20^ stated that PRF and CGF improve the inflammatory condition of the gingiva notably.

 This study evaluated the effect of PRF and CGF in the regeneration of soft tissue around dental implants. GEI-B decreased throughout the study, although it was not significant. GEI-P decreased in the control group and increased in the CGF and PRF group, which means that 24 months after surgery, the proximal embrasure around the implant was filled with gingiva in the PRF and CGF groups but not in the control group. These findings were not significant. Medikeri et al^19^ reported that 12 months after surgery, 91.7% of the implants in the PRF group had scored zero GEI, and the buccal gingival margin of the implant was at the level of the adjacent teeth. Inconsistent with this study, the study by Medikeri et al^19^ showed that PRF was significantly effective in improving the soft tissue around dental implants. In contrast to this study, Isler et al^20^ stated that the gingival recession around the implants in the CGF group increased from 0.04 mm to 0.25 mm. They concluded that CGF did not effectively prevent gingival recession. Qiao et al^21^ claimed that CGF was ineffective in preserving and reconstructing soft tissue around the dental implants. This difference may be due to different CGF protocol preparations. In the current study, CGF was prepared by centrifuging the blood samples for 2 minutes at 2700 rpm, 4 minutes at 2400 rpm, 4 minutes at 2700 rpm, and 3 minutes at 3000 rpm. In the study by Isler et al,^20^ samples were centrifuged for 2 minutes at 2700 rpm and 3 minutes at 3000 rpm. Qiao et al^21^ centrifuged the samples for 15 minutes at 4500 rpm.

 This study evaluated the effect of PRF and CGF in the regeneration of hard tissues around dental implants. The level of crestal bone to the implant platform increased significantly in all three study groups 24 months after surgery. The dimensions of periapical lesions decreased significantly in all the study groups. Medikeri et al^19^ showed that the level of crestal bone to the implant platform increased significantly 12 months after surgery in the PRF group (0.6 mm in the buccal, 0.48 mm in the lingual, 0.7 mm in the mesial, and 0.49 mm in the distal surface). Qiao et al^21^ reported that the crestal bone level was reconstructed around 3.7 mm in the CGF group one year after surgery. Manoj et al^22^ found that 6 months after the insertion of immediate implants with CGF, the bone level increased significantly by 2.3 mm on the buccal surface, 1.52 mm on the lingual surface, and 2.97 and 4.26 mm in the mesial and distal surfaces of the dental implants. Consistent with this study, Qiao et al^21^ and Manoj et al^22^ concluded that CGF enhanced the reconstruction of hard tissue around the dental implants. It can be concluded that PRF and CGF improved bone formation at the crestal level and the apex of the implant.

 The IHS showed optimum health in the PRF and CGF groups. In contrast, in the control group, IHS varied from optimum health to satisfactory survival and compromised survival during the two-year follow-up. In the study by Medikeri et al,^19^ the implant survival/success rate was 91.67% after one year in the PRF group.

 PRF and CGF play a pivotal role in bone regeneration by acting on several cellular and molecular mechanisms that enhance osteogenesis, angiogenesis, and tissue healing. These effects are mediated through the release of bioactive molecules, recruitment of specific cell types, and regulation of inflammatory responses.^24-26^

 PRF and CGF are both rich in growth factors such as platelet-derived growth factor (PDGF), transforming growth factor-beta (TGF-β), vascular endothelial growth factor (VEGF), and insulin-like growth factor-1 (IGF-1).^24,25^ These factors are released sustainably upon implantation and initiate a cascade of molecular signals essential for tissue repair. PDGF, in particular, plays a crucial role in recruiting mesenchymal stem cells (MSCs) to the site of injury, while TGF-β promotes the differentiation of these MSCs into osteoblasts.^26^ The release of VEGF from PRF and CGF promotes angiogenesis, creating a favorable environment for bone formation by enhancing vascularization and oxygen delivery. These growth factors work in synergy to enhance cell proliferation and the differentiation of osteoblasts, a process essential for the formation of new bone.^27-29^

 PRF enhances osteoblast differentiation by upregulating bone morphogenetic proteins (BMP-2 and BMP-7), which in turn stimulate the expression of Runx2, a critical transcription factor for osteoblast differentiation and bone matrix formation. This osteoinductive potential is vital for enhancing bone regeneration at the implant site.^28,30^ Furthermore, CGF provides a fibrin matrix that serves as a scaffold for osteoblasts to attach and proliferate, promoting mineralization and extracellular matrix deposition. The three-dimensional fibrin network of both PRF and CGF is rich in fibronectin and vitronectin, proteins that enhance cellular adhesion and the deposition of bone matrix. This network supports not only osteoblast differentiation but also accelerates bone healing through matrix synthesis.^27,29,30^

 Angiogenesis is a critical process for bone regeneration, and both PRF and CGF promote new blood vessel formation through the sustained release of VEGF. VEGF stimulates endothelial cells to proliferate and form new blood vessels, ensuring adequate nutrient and oxygen supply to the regenerating bone tissue.^25,28,31^ Furthermore, both PRF and CGF modulate hypoxia-inducible factor-1α (HIF-1α), a key regulator of the cellular response to low oxygen levels. HIF-1α activation increases VEGF production, further supporting angiogenesis and improving the survival of osteoblasts and other regenerative cells within the healing bone.^25,27,28^

 PRF and CGF promote osteogenesis and angiogenesis and play an essential role in modulating inflammation. The leukocyte-rich fibrin matrix of both PRF and CGF contains immune cells that release anti-inflammatory cytokines such as interleukin-10 (IL-10), which suppresses excessive inflammation and promotes tissue healing. The regulation of inflammation is crucial for preventing bone resorption and ensuring proper tissue regeneration.^32,33^ Additionally, both PRF and CGF inhibit osteoclastogenesis by reducing the expression of RANKL (receptor activator of nuclear factor kappa-Β ligand), which is involved in bone resorption. By modulating these inflammatory pathways, PRF and CGF help create an environment conducive to bone formation while minimizing bone loss.^31,33^

 One of the limitations of this study was that the current study did not include histological evaluation of bone regeneration or microbiological assessment of peri-implant conditions, which could provide a more detailed understanding of the healing process. Despite randomization, factors such as individual healing capacity, individual bone density, initial bone level, and oral hygiene maintenance were not assessed, which are among the limitations of this study. Future studies should focus on extending follow-up periods beyond 24 months to determine the long-term effects of PRF and CGF on implant survival, crestal bone levels, and soft tissue stability. Research should include patients with systemic conditions like diabetes or osteoporosis and high-risk groups such as smokers to determine the efficacy of these biomaterials in compromised healing. Comparative studies with other autologous or synthetic growth factors could help establish the most effective regenerative approach. Microbiological assessments could determine the impact of PRF and CGF on bacterial colonization around implants, and histological analysis may provide insights into bone and soft tissue quality. Further investigation into the cellular and molecular mechanisms of PRF and CGF, including their growth factor release kinetics and effects on bone remodeling, could enhance understanding of their regenerative potential.

## Conclusion

 It should be pointed out that active infection may interfere with osseointegration. However, if the remaining periapical infection is removed from the extraction socket of the teeth with periapical lesions, the immediate insertion of dental implants can be successful. PRF and CGF guarantee the successful osseointegration of immediate implants in previously infected sites. CGF had more promising effects than PRF. Also, after a two-year follow-up, the implant survival/success score showed optimum health for the CGF and PRF groups. PRF and CGF reduced postoperative pain, decreased BOP, improved the soft tissue regeneration around the implants, increased the crestal bone level, and reduced the periapical lesion size. It can be concluded that CGF and PRF positively affected the soft and hard tissues around the immediate implants placed in previously infected sites.

## Competing Interests

 No conflicts of interest.

## Ethical Approval

 The following approval code was obtained from Guilan University of Medical Sciences: IR.GUMS.REC.1399.461. The following approval code was obtained from the Iranian Registry of Clinical Trials: IRCT20180416039327N4.
